# Expertise Classification of Soccer Goalkeepers in Highly Dynamic Decision Tasks: A Deep Learning Approach for Temporal and Spatial Feature Recognition of Fixation Image Patch Sequences

**DOI:** 10.3389/fspor.2021.692526

**Published:** 2021-07-26

**Authors:** Benedikt Hosp, Florian Schultz, Enkelejda Kasneci, Oliver Höner

**Affiliations:** ^1^Human-Computer Interaction, University of Tübingen, Tübingen, Germany; ^2^Institute of Sports Science, University of Tübingen, Tübingen, Germany

**Keywords:** eye tracking, deep learning, convolutional neural network, long short-term memory, expertise, machine learning, football

## Abstract

The focus of expertise research moves constantly forward and includes cognitive factors, such as visual information perception and processing. In highly dynamic tasks, such as decision making in sports, these factors become more important to build a foundation for diagnostic systems and adaptive learning environments. Although most recent research focuses on behavioral features, the underlying cognitive mechanisms have been poorly understood, mainly due to a lack of adequate methods for the analysis of complex eye tracking data that goes beyond aggregated fixations and saccades. There are no consistent statements about specific perceptual features that explain expertise. However, these mechanisms are an important part of expertise, especially in decision making in sports games, as highly trained perceptual cognitive abilities can provide athletes with some advantage. We developed a deep learning approach that independently finds latent perceptual features in fixation image patches. It then derives expertise based solely on these fixation patches, which encompass the gaze behavior of athletes in an elaborately implemented virtual reality setup. We present a CNN-BiLSTM based model for expertise assessment in goalkeeper-specific decision tasks on initiating passes in build-up situations. The empirical validation demonstrated that our model has the ability to find valuable latent features that detect the expertise level of 33 athletes (novice, advanced, and expert) with 73.11% accuracy. This model is a first step in the direction of generalizable expertise recognition based on eye movements.

## 1. Introduction

In general, expertise research spans many different areas. Expertise research based on behavioral data has found its way into several fields, i.e., dentistry (Castner et al., 2020), surgery (Eivazi and Bednarik, 2011; Kübler et al., 2015; Hosp et al., 2021b), and sports (Discombe and Cotterill, 2015; Fegatelli et al., 2016; Kredel et al., 2017; Moran et al., 2018; Snegireva et al., 2018; Hosp et al., 2021a). In all of these areas, the assessment of user expertise is a fundamental task. By estimating the expertise of an user as accurately as possible, adaptive systems can be built to model different, distinct expertise classes and potentially create tasks specifically adapted to the expertise class. For diagnostics, within the framework of sports science expertise research, groups of different performance levels are examined using the “expert-novice paradigm” (Chi et al., 1981). According to Tenenbaum et al. (2000), this is the most efficient way to study the development of cognitive and motor skills. Based on this paradigm, Ericsson and Smith (1991) developed the frequently used framework of the “Expert Performance Approach”. This approach assumes that the behavior of a participant in a laboratory task is closest to their behavior on the pitch if the laboratory setting is as realistic as possible. It is, therefore, required to establish the highest possible ecological validity of laboratory tests, taking into account the internal validity (Kredel et al., 2017). According to this assumption, within the Expert Performance Approach, sports-specific scenes are often selected as stimuli for diagnostic (Romeas et al., 2016). However, in previous studies the video stimuli were mostly presented on large screens or PC monitors and often from a third-person perspective (for review, refer to e.g., Mann et al., 2007; Murr et al., 2020). This classical laboratory setting results in a low external validity (Marasso et al., 2014; for an overview refer to Travassos et al., 2013). The trade-off of these validities plays an important role. Especially in highly dynamic environments it is difficult to obtain robust and natural data. Robust data is obtained in highly controlled environments, while natural data is obtained in natural environments. Therefore, these two aspects are opposites and relative to discussions about the tension between the internal and the ecological validity of scientific studies. This is especially true in fields such as sports, where besides tactical and physical components, highly refined perceptual-cognitive abilities are key to success (Helsen and Starkes, 1999; Berry et al., 2008; Catteeuw et al., 2009; Abernethy, 2010). Since the physical strain of the athletes in high-level sports is very high due to training several times a day, enhancing cognitive factors like decision-making without additional physical training is gaining in importance (Appelbaum and Erickson, 2016). For this reason, research efforts to identify the major cognitive factors leading to differences in performance, especially with regard to decision-making in the sports game have increased in recent years. One aim of these efforts is the development of valid diagnostics that can, for example, identify the gaze behavior of experts engaged in successful decision-making. Accordingly, by teaching this gaze behavior it may be possible to design training programs that lead to improved decision-making.

Due to ongoing technological development in the field of virtual reality (VR), it is now possible to present 360° stimuli from a first-person perspective in head-mounted displays (HMD). This increases the feeling of “presence” for participants, which is defined as the psychological experience of “being there” (Cummings and Bailenson, 2016). An increased feeling of presence should lead to more valid results as compared with presentations on screens (Slater, 2018; Bird, 2019). In addition to the valid stimulation and recording of behavior, an analysis of the underlying mechanisms of expertise is necessary to formulate explanatory approaches for identified performance differences. For the analyses of cognitive processes (e.g., decision-making under pressure or anticipation of the continuation of a scene) in sport games, new developments in image processing, measurement methods, machine learning and eye tracking may be used to control the stimuli or utilized as non-invasive methods that do not influence the natural behavior of the athlete. The developments in eye tracking have shown that these methods of measurement hardly disturb natural behavior, but, instead, become increasingly accurate and informative because cognitive processes, such as perception are very simple, non invasive, and meaningful to track.

In sports science, the non invasive method of eye tracking is considered a common and objective research method for the analysis of visual attention and the intake of visual information (for an overview refer to Hagemann et al., 2006). In this study, it is also assumed that the measurement of athlete gaze behavior in real sports situations generates the highest ecological validity. Mobile eye trackers have disadvantages (e.g., inaccurate measurements due to slippage or low frequencies), that can be circumvented by eye trackers integrated into the HMD. Due to the 360° videos that can be presented there, gaze behavior can be recorded at high frequency (up to 250 Hz) in ecological valid environments with high experimental control.

The type of analysis also plays an important role because up until this point eye tracking data has mainly been evaluated manually, visually, or with statistical methods (Blascheck et al., 2017). A newer and popular technique to classify expertise is to train a model by a brute-force approach of all possible features available from the data. Hosp et al. (2021a) use this technique to investigate the expertise of soccer goalkeepers by recording their gaze during game build-up. In their approach to expertise recognition, they take all possible features provided by the eye tracking vendor and add derived statistical features on top. They found a support vector model (SVM) with high accuracy. However, this feature crafting is highly time-consuming and does not necessarily provide the most suited features. There is no real evidence that certain features or feature combinations highlight expertise. For example, Klostermann and Moeinirad (2020) revealed that fixations, saccades, and their frequencies and lengths are often used, but cannot lead to a full understanding of expertise. They conclude that single features describing gaze behavior are only conditionally suitable to classify expertise differences or, at they very least, have yet to be found. Relatively, expertise comes from the optimized perception of helpful gaze locations and the sequence of these locations, which are also called scan path. To explore the gaze locations and their temporal succession, the approach is to let an artificial intelligence (AI) describe the features around these gaze locations (albeit very abstract). In doing so, the AI itself decides which shapes, colors, corners, and edges in the fixation locations are considered important for distinguishing expertise. This does not lead to new insights about the features of gaze behavior in athletes. However, the sequence of fixation locations from the stimulus can be used, first to automatically recognize expertise and differences in the scan path, and second, given sufficient data, to generate an optimal scan path. Ultimately, this scan path, can help one understand important expertise-related fixation locations and their sequence in the gaze signal. Furthermore, with an optimal scan path one can infer the importance of opponents, teammates (or at least parts of such), or the ball for the decision-making process. By looking at the fixation patches and running an object or person detection, a successful orientation of the scene can be achieved.

This leads to a large amount of data which is advantageous for machine learning as machine learning algorithms show their strengths in regression and the classification of large amounts of data. Even in supervised machine learning algorithms, where features need to be selected first, we often face the problem of choosing optimal features because there is no indication as to which set of features can best show the expertise of a class.

Next to supervised learning there are other approaches that work in an end-to-end learning fashion, where features do not need to be identified beforehand. The most important representatives in this field are the convolutional neural networks (CNNs) and recurrent neural networks (RNN), i.e., bidirectional long short-term memory networks (BiLSTM). CNNs are well used in a range of applications including semantic segmentation and object recognition and can learn to distinguish relevant patterns and shapes or to derive abstract objects.

RNNs and particularly long short-term memory networks (LSTMs) (Hochreiter and Schmidhuber, 1997), which have the ability to find temporal relationships (Liu and Han, 2018; Tian et al., 2019), are also widely used. LSTMs optimize RNNs by minimizing the impact of vanishing and exploding gradients. By using a special function block, LSTMs implement a long short-term memory, which pushes the performance of neural networks. These functional blocks allow for the remembrance of long-time dependencies and previous information. The network learns which information from the past is important for the current output and which can be forgotten (by a forget gate). As the gaze signal is a continuous signal, LSTM are predesignated to be used in the analysis of temporal patterns in the gaze signal. Currently, both kinds of machine learning techniques are well used for expertise identification in different domains, e.g., in dentistry education (Castner et al., 2018, 2020), or microsurgery (Eivazi and Bednarik, 2011; Eivazi et al., 2012, 2017; Bednarik et al., 2013). Neural networks (Castner et al., 2020) and supervised learning algorithms (Bednarik et al., 2013; Castner et al., 2018; Hosp et al., 2021a,b) have both shown their power in objective expertise identification based on gaze behavior. They found major differences in gaze behavior and could link these differences to different expertise classes. This means that both machine learning techniques provide suitable methods to deal with large amounts of data and analysis in a fast, objective, and reproducible way.

To evaluate gazePatchNet, we conducted a study where we showed participants 360° stimuli of defined soccer game situations from the natural perspective of a goalkeeper on a consumer grade HTC Vive HMD. Each stimulus presented a build-up scene and ended after a pass to the user. The user's gaze was recorded by the integrated SensoMotoric Instruments (SMI) eye tracker with a frequency of 250 Hz. We used the eye tracking data to classify the expertise of the participants into three classes, namely, novice, advanced, and expert. This approach is meant to serve as a first step in the direction of a perceptual-cognitive training system. If our model is robust enough, the discovered knowledge can be used to identify optimal synthetic scan paths that can then be used to train the gaze behavior of athletes. The underlying hypothesis is that an improved gaze strategy leads to a more reliable recognition of cues and to better decision-making based on these cues.

## 2. Materials and Methods

### 2.1. Stimulus

To show the stimulus video material in virtual reality, we used the SteamVR framework prefab in Unity. SteamVR is an open-source framework that allows common real-time game engines, like Unity, to interface with HMDs. On contrary of an artificial recreation of the environment (simulation) within the game engine, we projected realistic footage of 4k omnidirectional videos that captured the inside of a sphere around the participant (3,840 × 1,920 pixel). This allowed us to display a natural stimulus with high immersion in a realistically mimicked scene. The 360° footage was captured in cooperation with the German Football Association (DFB) at the training space of the elite youth academy of a German first league club (VfB Stuttgart). To capture the footage, we placed a 360° camera at the position of the goalkeeper, while five teammates and five opponents were physically replaying the defined scenarios on the training space. The camera captured the scene with 30 FPS. Each scene was developed based on common scenarios during a match, each with unique movements. All stimuli were captured on-field and acted out by youth elite players of the highest level in Germany. At the end of each scene six options (five teammates to pass the ball or kick-out) occurred how to continue the game (see Figure 2 for an overview of the options). In each video, there was one optimal option leading to binary answers in each video, i.e., the optimal option was counted as one, all five other options were counted as zero. Plausibility of the scenes, movements, and ratings of the decision options were evaluated by an expert trainer team of the DFB.

### 2.2. Data Collection

The responses of participants were relayed verbally and finally rated as either right or wrong, with only one right decision available per video. The correct decision is a pass to the only teammate who is not covered by an opponent. In total, each participant saw 52 trials, consisting of 26 videos with unique movements, repeated in a different order. Each video trial of each participant was counted as one sample.

### 2.3. Participants

Characteristics of all participants are shown in Table 1. Data of (*n* = 12) experts were collected during a DFB goalkeeper camp, where the DFB gathers the top German soccer goalkeepers (U-15 to U-21) for specialized training. The data of the (*n* = 8) advanced and the data of (*n* = 13) novice athletes were collected in the lab of the university. The advanced players belong to different soccer teams playing in the southern regional league (semi-professional, 4th level) in Germany. The novices have very little to no experience in amateur leagues, up to district league, with no participation in competitions and no training on a weekly basis.

**Table 1:** Participants summary.

**Class**	**Age (Mean/SD)**	**Training hours/week (Mean/SD)**	**Active years (Mean/SD)**
Novice (*n* =13)	28.64 / 3.72	0.00 / 0.00	1.78 / 5.21
Advanced (*n* = 8)	22.00 / 3.72	4.94 / 0.91	15.50 / 5.77
Expert (*n* = 12)	16.60 / 1.54	8.83 / 4.27	9.16 / 5.04

### 2.4. Procedure

**Figure 1:**
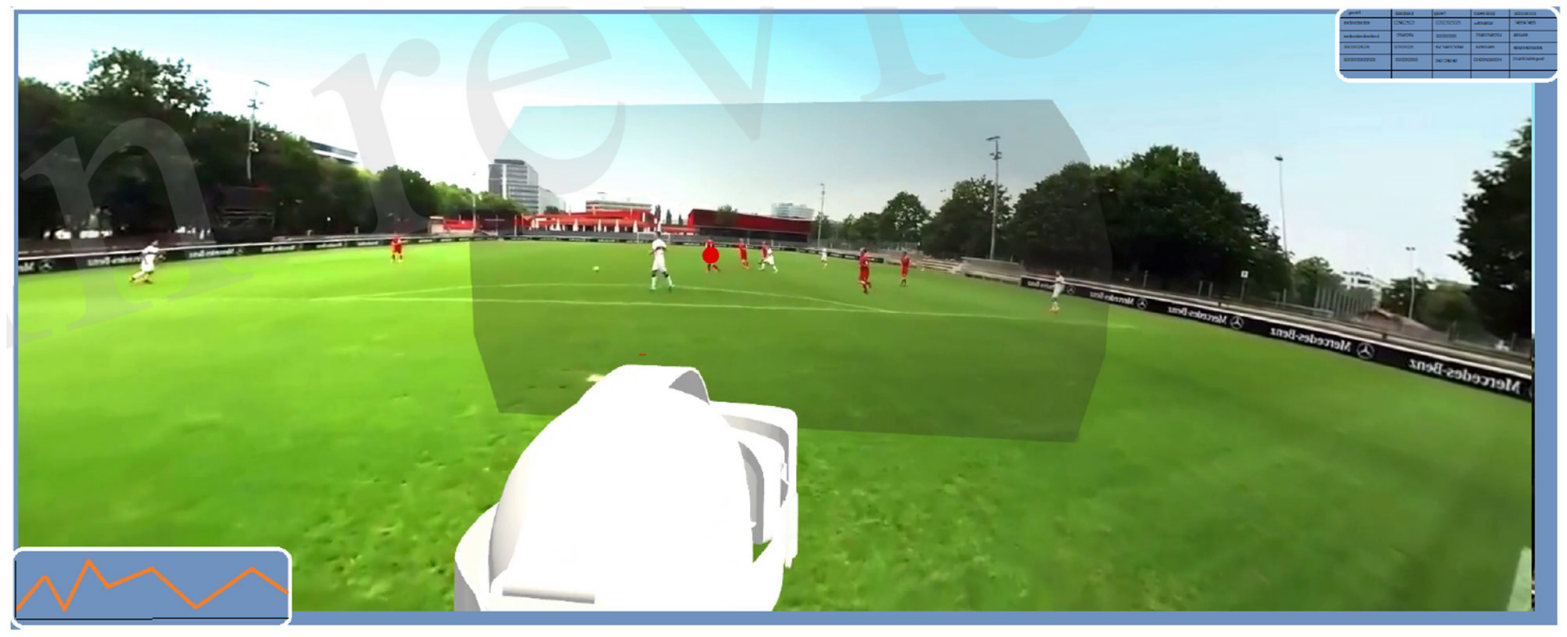
Example field of view in virtual reality head-set.

**Figure 2:**
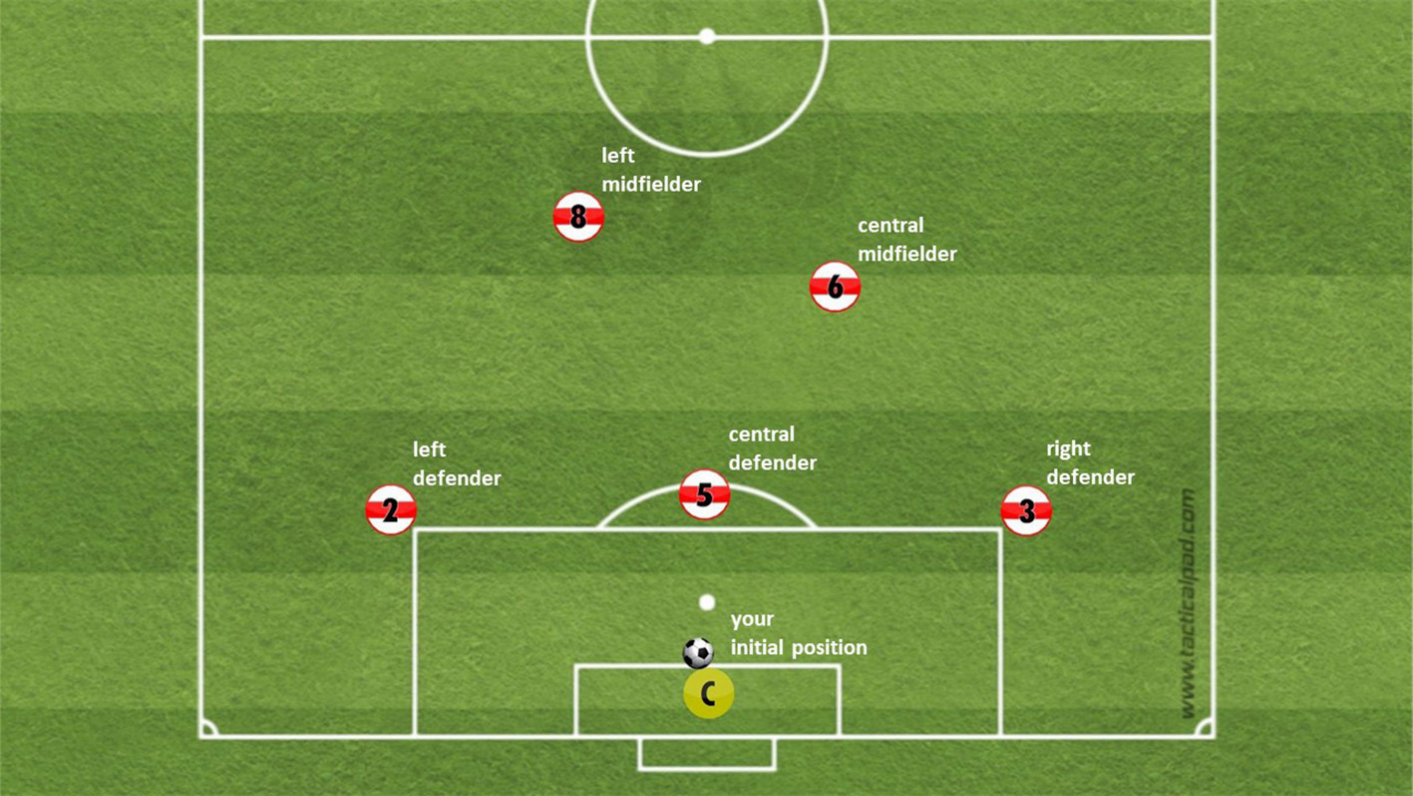
Schematic overview of the response options. The sixth option (kick out) is missing.

The study was confirmed by the Faculty of Economics and Social Sciences Ethic Committee of the University of Tübingen. After completing a consent form, we started familiarizing the participants with the stimulus presentation and response mode. During the familiarization phase, we showed a sample 360° screenshot of a video on the HMD to allow free exploration of the scene, followed by a schematic overview of the field (refer to Figure 2). After that, the video scene (refer to Figure 1 for an example) was played and stopped (black screen) after the last return pass to the position of the participant. In each scene we manipulated the color of the ball with a colored dot during the last return pass. This was done to force the gaze of the participants on the ball during this important phase. As soon as the screen went black, the participant had to report the color of the ball and their decision for an option to continue the game. The decision selection is identical to the initial schematic overview of the field (Figure 2) with an emergency option to “kick out.” This procedure was repeated five times.

After this familiarization phase, we started the first block of 26 trials. The second block contained the same 26 videos but in different order. Between the blocks, participants could take off the HMD for a break. The video in the Supplement Materials shows an example visualization of the setup in VR.

### 2.5. Image Patch Extraction

**Figure 3:**
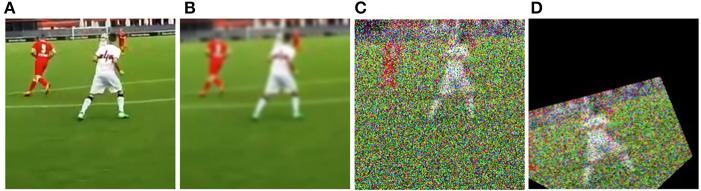
Augmentation pipeline. **(A)** shows the original image cut around a fixation, **(B)** shows the image after gaussian blur, **(C)** shows the image after salt and pepper noise addition, and **(D)** shows the transformed image.

As introduced above, this method (coined gazePatchNet) includes the following: (1) finding latent features in the image patches around the fixations of the participants and (2) classifying the scanpath as a sequence of the consecutive fixation image patches. The whole process is illustrated in Figure 3. As not all data collection went smoothly, because of slippage of the head-set (too loose) or bad calibration results, we reviewed the gaze signal quality of all samples. We considered a sample valid only if the tracking ratio was higher than 75%. We assigned either class 0 (for novice samples), class 1 (for advanced samples) or class 2 (for expert samples), to each sample. After removing invalid data points, we collected all gaze signal samples for each fixation (timestamp x and y) and saved them with the corresponding omnidirectional video file. The fixations were calculated with the velocity threshold-based event detection filter (I-VT) (Salvucci and Goldberg, 2000) algorithm of the vendor using a threshold of 50°/s. We calculated the temporal as well as spatial center of the fixation based on the averaged gaze signal samples of the fixation. Afterward, we looped over the video file frames to find the corresponding frame by timestamp and cut out an image patch around the fixation on the frame. The size of the patch fits the input size of the input layer of the GoogLeNet CNN (224 × 224 × 3 pixels), which we used to extract features later. As soon as we had all the fixation image patches of one trial, we created sequences that fit to the BiLSTM. These sequences were essentially fixation image patches in order of their occurrence in the stimulus video.

### 2.6. Data Augmentation

**Figure 4:**
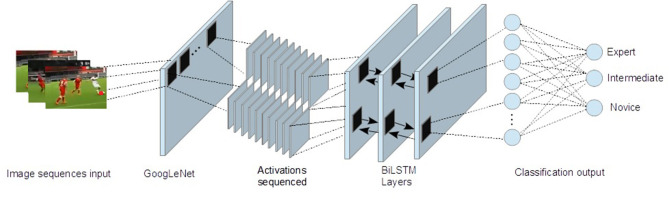
gazePatchNet: Our CNN-BiLSTM-based model architecture for expertise classification.

For each sequence, we computed a new and modified sequence containing the same images. This means we doubled the whole data set by adding the same sequences with the same, but augmented images. An example is shown in Figure 4. Figure 4A shows an input image (image cut from the stimulus around a fixation). At first, we applied a random Gaussian blur (Figure 4B) and salt and pepper noise (Figure 4C). Afterwards, we augmented the images in a randomized manner with geometric transformations (Shorten and Khoshgoftaar, 2019) (Figure 4D). Each image was either rotated by a random factor between -180 and 180 degrees, sheared by a random factor between –15 and 15 degrees or both, flipped on x- or y-axis or was x- or y- translated between –80 and 80 degrees. These augmentation steps were supposed to make training harder for the model in a realistic way. We assumed shear and rotation were real translational variations of the head of the participants (whole field of view around fixation). This data augmentation was completed before training in an offline manner. The whole data set was augmented in 135 s. LSTMs usually support varying sequence lengths. However, sequences that are much longer than typical sequences can introduce a lot of padding or discarding of data because of the padding or truncation of sequences. Thus, we removed an average of 20 sequences, about 2% of all sequences. The remaining sequences were sorted by length. This led to a more homogeneous padding of the input sequences.

### 2.7. Transfer Learning

**Figure 5:**
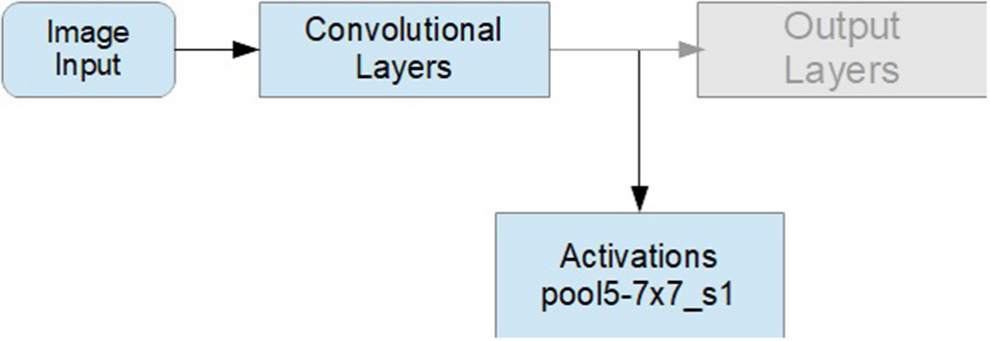
Transfer learning for feature selection.

To get latent spatial features in the image patches automatically, we used a CNN (GoogLeNet) as a feature extractor. The CNN was trained on ImageNet, which has about 1,000 classes. Each sequence (the augmented sequences included) was fed to the CNN. We did not use the output layers, as we did not need the classification probabilities for the 1,000 classes of ImageNet, but, rather, for three classes of expertise. On the contrary, we proceeded with transfer learning by grabbing the output of the last activation function [Refer to Figure 5, the last pooling layer of the GoogLeNet network (“*pool*5–7 × 7_*s*1_”)], and added the layers of gazePatchNet (Refer to Table 2). By using GoogLeNet as a feature extractor, we simultaneously obtained a feature dimension reduction. Thus, our images of 224 × 224 × 3 pixels were reduced by the CNN to 1,024 × 1 dimensions. As a result, we achieved not only shape, pattern, and object detection but also the correct input format for an LSTM by keeping track of the input to the CNN and building sequences of related outputs (activated images).

**Table 2:** GazePatchNet architecture.

	**Name**	**Type**	**Activations**
1	sequence	Sequence Input	1,024
2	bilstm	BiLSTM	1,000
3	fc-1	Fully Connected	100
4	dropout	Dropout	100
5	fc-2	Fully Connected	3

### 2.8. Training and Testing

We trained the model in 33 runs. In each run, the samples of one participant were kept out (leave-one-out validation). The sequences of this participant were used at the end of each run to predict their class. As the model has not seen the samples of the one subject which was left out, these samples can be used to show the predictive power and classification accuracy of the trained model on unknown data. The data of the remaining 32 participants was split by a ratio of 70:30. About 30% of the data were randomly picked for testing and optimization during training. The remaining 70% of the samples (as well as the augmented samples) were used for training the model.

### 2.9. Model Description

Table 2 shows the structure of the layers of the networks. The sequenced activations, containing the selected features, from GoogLeNet were passed to the BiLSTM layers, where the temporal relationships were calculated. To input sequences of images into the network, the first layer was a sequence input layer with the same input dimensions (1,024) as the output of the activations by the CNN at the last pooling layer (GoogLeNet). As the models with gated recurrent units (GRU) and LSTM layers did not perform well in our tests (between 20 and 25% lower accuracy), we chose BiLSTM layers as the next part. The BiLSTM layer had 50 hidden units (therefore 4,000 × 1,024 input weights, 4,000 × 500 recurrent weights and 4,000 × 1 biases) and output only the final step. The advantage of BiLSTM layers is that they are fairly generative and take future (forward) and past (backward) states of information into account. After the BiLSTM layer, we added a fully connected layer with 13 hidden units (100 × 1,000 weights and 100 × 1 biases). To prevent the model from over fitting, we added a dropout layer with a probability of not using a neuron of 50%. As we had three classes to predict, the following fully connected layer had three hidden units. We took the maximum output to identify the class. To help training converge quickly, we added a softmax layer and calculated the cross-entropy loss for multi-class classification to optimize the model.

Table 3 provides an overview of the training options. We used grid search to find an optimal hyper parameter set for the whole network (Feurer and Hutter, 2019). The best set consisted of a mini-batch size of 42, a low learning rate of 4.4e-4, which was not increasing during training time, a L2-regularization of 8.2e-4, to prevent over-fitting, and a validation frequency that was set to 52, so that the model was validated at every epoch. A validation patience of 6 seemed to be the optimal trade-off between over and under fitting. This means that the training was stopped earlier if loss on the validation set was larger than or equal to the previous smallest loss of six times in a row. We did not shuffle training and validation data every epoch, as we only wanted to use validation data to offer information about the current classification status. The maximum number of epochs for training was set to 100, as longer training results in over or under fitting.

**Table 3:** Training options.

**Parameter**	**Value**
MiniBatch size	42
Learning rate	4.4e-4
L2-Regularization	8.2e-4
Sequence length	longest sample
Shuffle	no
Validation frequency	52
Validation patience	6
Learning rate schedule	no
Max. epochs	100

### 2.10. Metrics

We calculated the average/median accuracy of all runs. In each run, 70% of the samples belonged to training set and 30% to the validation set. We kept one participant totally out to test how well the model behaved on new, unseen data. As accuracy is a metric defined by the number of correct predictions divided by the total number of predictions, we could only infer a small amount of information about the model. This was particularly because the samples of the classes used for training and validation were balanced during trainings, but the distribution of expert, advanced, and novice participants for testing was not. Thus, we also had to consider further performance metrics of the model. The confusion matrix is a sound metric to show true and false positives of the single classes. Similar to the confusion matrix, the following metrics were split into the three classes for easy comparison. To gain a deeper performance insight, we showed the receiver operating characteristic (ROC) curve. An ROC curve shows the performance of a classification model at different classification thresholds. Based on the ROC curve, we simply calculated the area under the curve (AUC), which is a common single score and is used for comparisons between different models, usually on a binary classification. Since we split the classes and computed the AUC for each, we compared which classes were predicted most successfully. A score of 1.0 described a perfect skilled model. All scores were calculated by an one-vs-all approach.

## 3. Results

**Figure 6:**
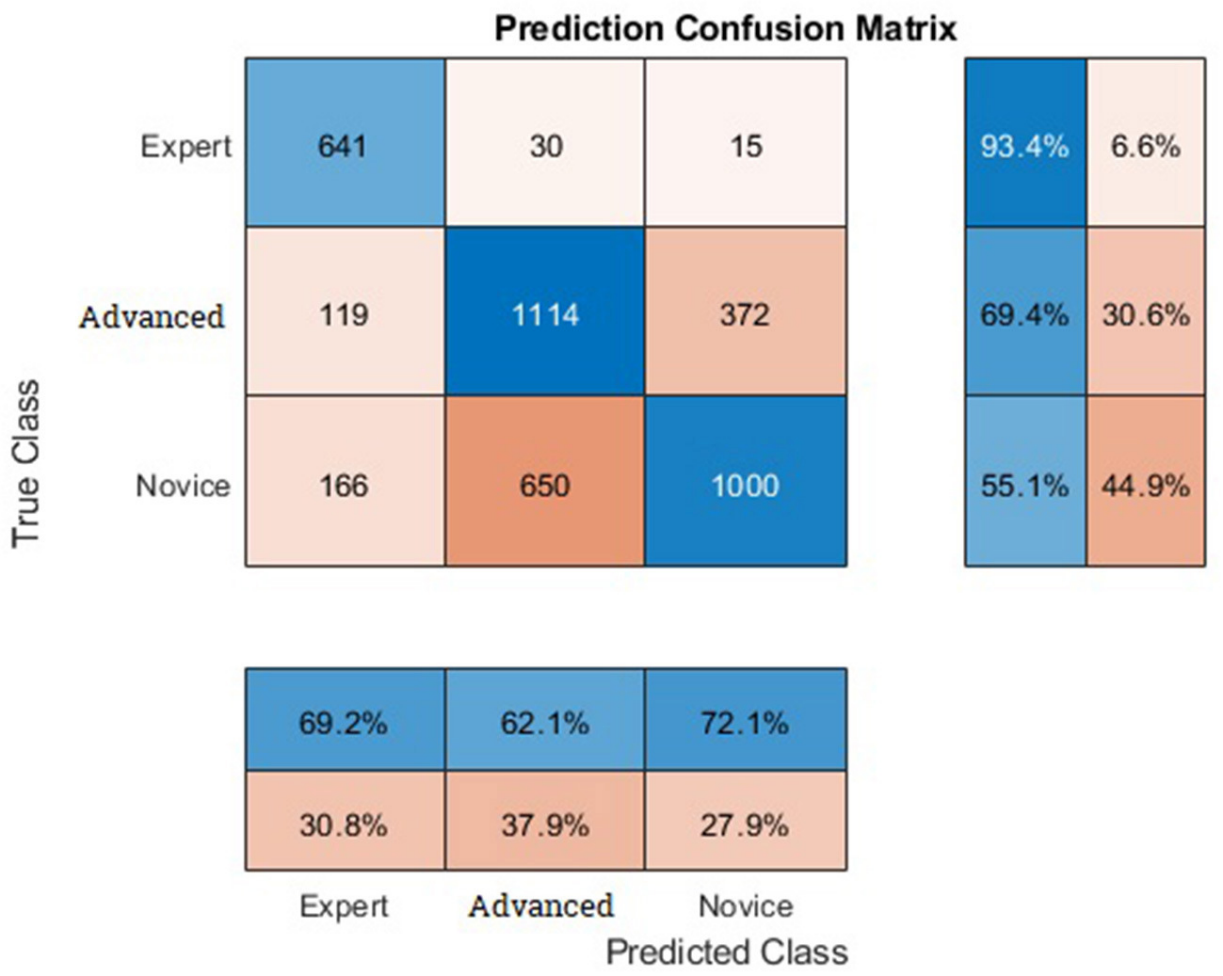
Confusion matrix.

The model achieved an average accuracy of 73.11% over 33 runs. For each run, data from one participant were kept out of training and used as test data. We looked at the data indirectly by describing one trial (one video of a participant) as one sample and classifying these samples as novice, advanced, or expert. This means that some participant samples can be detected as belonging to another group. The distributions of the single samples to different classes is shown on the confusion matrix in Figure 6. The accuracy of predicting a novice correctly is at 55.1%. The prediction rate of the advanced class with an accuracy of 69.4%, is admittedly much higher. And even higher than the advanced class, experts are predicted with an accuracy of 93.4%.

Out of 1,816 samples of the novice class, 166 samples were predicted as belonging to the expert class and 650 to the advanced class. 1,114 samples were correctly classified as advanced. However, about one-third of the advanced samples were predicted falsely, which is distributed as 372 on novice class and 119 on expert class. About 641 of the expert samples were correctly predicted and 30 samples to advanced and 15 to novice class.

**Figure 7:**
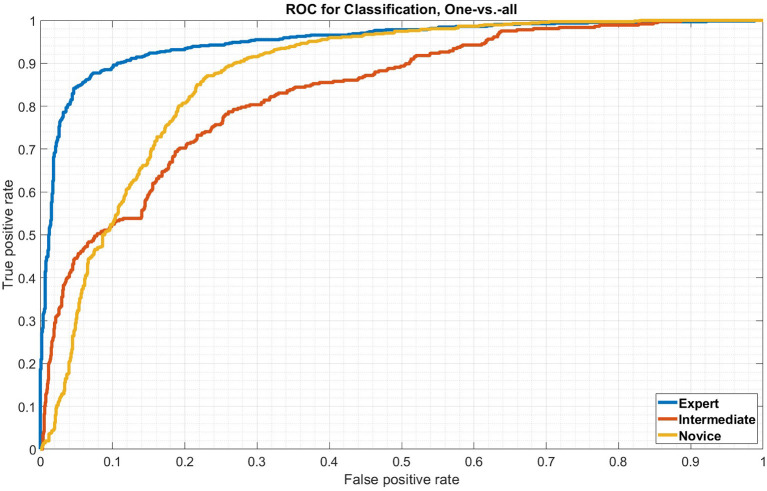
ROC-curve for all three classes.

Figure 7 shows three ROC curves with results, corresponding to the confusion matrix. The blue line represents the expert ROC curve. With an AUC of 0.951, the classification is nearly perfect. This corresponds to the confusion matrix values, as well. The red line represents the advanced class which does not perform as well as the expert, but still achieves an AUC of 0.833. The yellow line shows the performance of the novice class, which is a little bit higher than the advanced, with an AUC of 0.871.

## 4. Discussion and Conclusion

In this study, we presented gazePatchNet, a model that, based on by-transfer learning, adapted CNN for feature extraction and BiLSTM for temporal dependency identification, automating classification in the broadest sense. We recorded the gaze behavior of soccer goalkeepers during build up in a 360° video environment on a HMD and used their fixation image patches on the stimuli as input signals to classify three groups of expertise. The results are promising as we can show, with a relatively small amount of data, that the combination of a CNN, transfer learning, and BiLSTM network is effective in classifying this kind of data. At least the expert and advanced classes are clearly recognizable. However, the novices look more diverse in their behavior and therefore are much harder to predict. The model on average shows great performance, which is reflected by the average accuracy of 73.11% with great sensitivity values visible in the ROC plot. The differences between expert and the other groups are especially significant.

The classification of the advanced and novice class is about 20 and 40%, respectively, lower, but advanced are still doubled when compared with chance-level. This is supposed to increase with more samples for the model to learn from. These results are well in line with other studies on dynamic tasks, e.g., Bednarik et al. (2013) or Eivazi et al. (2017) who reached a classification accuracy of 66 and 70%, respectively, on medical applications. Both studies predicted the expertise of two skill levels. A more diverse result is found in Castner et al. (2018). Their study predicted the expertise of students from five different semesters alongside experts. With their one-vs-all approach, they mostly reached an accuracy of 37% (chance-level 20%).

In this model, the accuracy of predicting an expert correctly is at 93.4% as this class is the easiest to detect. The prediction rate of the advanced class is much lower with an accuracy of 69.4% because this class is supposed to be the hardest to detect. The accuracy, however, is nearly double the chance-level with about two-third of the advanced samples being classified correctly. Much lower than the advanced, the novices are predicted with an accuracy of 55.1% which is nearly two times as high as the chance-level but still 15% lower.

Out of 686 samples of the expert class, only 15 were predicted as belonging to the novice class and 30 to the advanced class. 1,114 samples were correctly classified as advanced, but about one-third of the advanced samples were predicted incorrectly. Although interesting, this is no surprise. It may show that the decision boundary for the advanced class does not need to be so robust as many of the advanced participants were gazing like novices and many novices as advanced according to the model. In summary of the performance of the classifications based on the ROC curves, one can state that the samples of all classes were predicted with high certainties and demonstrate the accuracy of a highly skilled predictive model. The average precision value (73.11%) and the mean precision of 71.89% confirm the power of the model.

Looking deeper at the ROC-curves, the model performs well on all classes. As samples of advanced players are often predicted as belonging to an novice class and samples of novices players as belonging to an advanced class, it may be necessary to increase the sample size, to robust the decision boundary. In case the model predicts a sample that really belongs to the expert class, it performs this assignment with a high probability of over 93%.

In this study, the novice and advanced classes are more difficult to classify. This means that the expert group is a pretty well recognizable group. The advanced and novice groups are more heterogeneous as there are participants that have more/less experience than others. Another reason for this could be the missing metrics needed to divide between the two classes properly. This question is typically addressed with the availability of more data. The problem may stem from the small sample size of advanced participants as this group could be too small for the model to define robust decision boundaries. The fact, that expert samples were barely (15 samples) predicted to be samples of an advanced player, shows that there are clear decision boundaries for the advanced and expert classes. In addition, the cognitive factor is only one of the several factors that contribute to expertise. For goalkeepers, for example, it is still most important to be able to block shots on goal. If a goalkeeper can do this extremely well, he may be invited by the DFB, even though he could make “worse” decisions after return passes. Conversely, it can also be the case with advanced participants are very good decision-makers, but they did not hold as many balls, which is why they are not invited by the DFB. As a result, it is very important to not just test players from different classes but to test players with the assumed highest decision-making skills. For the diagnosis of expertise, we aim to test the best of the very best players and compare them with other expertise groups. We need them to define an optimal behavior. The expert players are among the 50 most successful young goalkeepers in Germany, which is reflected in the results of this model. A long range plan is to optimize the training for young players. This study is the first step in that direction. For that, we need to know which behavior is optimal and how we can design training steps for young players to reach this optimal behavior.

The difference in active years/training, and therefore experience, between advanced and expert participants is much smaller and needs to be finer graded. There may be advanced players with a lot of experience that helps them to perform like experts and there may be experts that do not perform as well because they have much less experience. It is, therefore, not astonishing that some advanced samples are recognized as expert samples. If one assumes that behavior in some samples is better than others, this consequently leads to classifications distributed in different groups. It is more important that the number of classifications of higher ranked participants into lower classes is minimized to depict real expertise.

Instead of providing a description of the behavior of different classes, this model describes a pipeline to find latent features by itself. This circumvents one problem: handcrafted features. The characteristics of handcrafted features may be difficult to teach a user in the form of new behavior based on feature values. Even if the optimal set of features is found, it is difficult to incorporate the findings into a training system. For future, this model offers a different way of teaching a participant a new behavior by visualizing the test person what has when to be observed. Therefore, a model should be created that in the best case, finds an optimal behavior. Based on such information, an optimal behavior for each class can be created and artificially extracted to create information that can be taught to users. A prerequisite will be the analysis of single scan paths, which can be accessed by looking at the fixation image patches.

Currently, as the fixation point is temporally and spatially averaged, another improvement might be achieved when optimizing the input layer by using an object detection beforehand. Especially when counting in the error rate of the eye tracker and early fixations some samples might end up directly next to an object and some directly on it. In this case, the CNN will return different shapes. By using the object as an area of interest (AOI) and taking the intersection as input, this behavior can be unified as one can assume that the participant is perceiving the same object in both cases. The CNN can also be optimized. At the moment this CNN is trained on ImageNet to classify about 1,000 classes. By retraining the CNN on a set of 360° videos, with manually labeled teammates, opponents, goals, ball, and free spaces, the intersections of the gaze with AOIs can become advantageous and result in higher classification rates.

## 5. Perspectives

As aforementioned, the results already allow for the use of this model as a diagnostic system and as the basis for a training system. The information gathered from this study can be used to model behavior of athletes to personalize new adaptive interfaces that can understand user behaviors based on relevant user information recorded during training. For example, Wade et al. (2016) performed intervention for individuals with autism spectrum disorders.

With an objective way to classify the perceptual skill of a person, a first step toward a virtual reality training system (VRTS) with an adaptive design of level difficulty is achieved. With a definition of the perceptual skill of a person and the knowledge of the corresponding skill class, the choice of the difficulty level in a VRTS can be adapted automatically. For higher ranked users, the difficulty can be increased by pointing out fewer cues or adapting the stimulus, e.g., by placing relevant information outside the foveal area (usage of peripheral vision), designing more crowded scenes (retain overview) or showing highly dynamic situations (faster perception and reaction times). A fundamental challenge for such a VRTS is to enhance the model with more classes and more participants per each class. More data need to be collected to create a more robust model. A balanced data set would reveal interesting effects on recall and precision and, based on the current performance, might even increase the overall accuracy as the class with the least number of samples has the highest precision values. Different kinds of models also need to be investigated. For feature extraction, a network which is trained on human detection might provide even better results as the head/face and other parts of the human anatomy are potentially considered to be of importance. With 33 participants and an average accuracy of 73.11% on the test set, this model is suitable to be used for this kind of classification.

In a further step, to research the applicability of this model, we need to focus on adequate training scenarios. The system can, for example, be used to create an optimal synthetic scan path. By using the knowledge, discovered by this model, one can implement a generative adversarial network. This technique learns to generate new data, in this case a new scan path, with the same statistics as this training set. With enough data to train gazePatchNet to provide strong robust classes, a synthesized optimal scan path can be created. Should this be possible, it could also become relevant from a practical sports perspective to teach a certain gaze strategy obtained from the generative model. The optimal scan paths identified for each scene could be used to train the gaze behavior of athletes. The underlying hypothesis is that an improved gaze strategy leads to a more reliable recognition of cues and better decision-making based on these cues. To investigate this, however, appropriate training studies are necessary, which must provide information as to (a) whether it is feasible for athletes to replace their gaze behavior, developed over years, with a foreign behavior and, if so, (b) whether the modification of their gaze behavior also leads to better decision-making in the lab. Finally, the possibility of a corresponding transfer to the field must be checked.

## Data Availability Statement

The datasets presented in this study can be found in online repositories. The names of the repository/repositories and accession number(s) can be found here: https://atreus.informatik.uni-tuebingen.de/hosp/goalkeeperexpertisesupvervisedml_data.

## Ethics Statement

The studies involving human participants were reviewed and approved by Faculty of Economics and Social Sciences Ethics Committee of the University of Tübingen. Written informed consent to participate in this study was provided by the participants' legal guardian/next of kin. Written informed consent was obtained from the individual(s), and minor(s)' legal guardian/next of kin, for the publication of any potentially identifiable images or data included in this article.

## Author Contributions

BH: writing, analysis, coding, conception, and study. EK: writing and supervision. FS: conduction of study, material, and writing. OH: conduction of study, material, writing, and funding acquisition. All authors contributed to the article and approved the submitted version.

## Conflict of Interest

The authors declare that the research was conducted in the absence of any commercial or financial relationships that could be construed as a potential conflict of interest.

## Publisher's Note

All claims expressed in this article are solely those of the authors and do not necessarily represent those of their affiliated organizations, or those of the publisher, the editors and the reviewers. Any product that may be evaluated in this article, or claim that may be made by its manufacturer, is not guaranteed or endorsed by the publisher.
